# Mitigation of Memory Impairment with Fermented Fucoidan and λ-Carrageenan Supplementation through Modulating the Gut Microbiota and Their Metagenome Function in Hippocampal Amyloid-β Infused Rats

**DOI:** 10.3390/cells11152301

**Published:** 2022-07-26

**Authors:** Ting Zhang, Xuangao Wu, Heng Yuan, Shaokai Huang, Sunmin Park

**Affiliations:** 1 Department of Bioconvergence System, Hoseo University, Asan 31499, Korea; zhangting92925@gmail.com (T.Z.); niyani0@naver.com (X.W.); yuanheng.changan@gmail.com (H.Y.); huangsk0606@gmail.com (S.H.); 2 Department of Food and Nutrition, Obesity/Diabetes Research Center, Hoseo University, Asan 31499, Korea

**Keywords:** fucoidan, λ-carrageenan, Alzheimer’s disease, short-chain fatty acids, fermentation

## Abstract

Attenuating acetylcholinesterase and insulin/insulin-like growth factor-1 signaling in the hippocampus is associated with Alzheimer’s disease (AD) development. Fucoidan and carrageenan are brown and red algae, respectively, with potent antibacterial, anti-inflammatory, antioxidant and antiviral activities. This study examined how low-molecular-weight (MW) and high-MW fucoidan and λ-carrageenan would improve memory impairment in Alzheimer’s disease-induced rats caused by an infusion of toxic amyloid-β(Aβ). Fucoidan and λ-carrageenan were dissected into low-MW by *Luteolibacter algae* and *Pseudoalteromonas carrageenovora*. Rats receiving an Aβ(25–35) infusion in the CA1 region of the hippocampus were fed dextrin (AD-Con), 1% high-MW fucoidan (AD-F-H), 1% low-MW fucoidan (AD-F-L), 1% high-MW λ-carrageenan (AD-C-H), and 1% low-MW λ-carrageenan (AD-C-L) for six weeks. Rats to receive saline infusion (Normal-Con) had an AD-Con diet. The AD-F-L group showed an improved memory function, which manifested as an enhanced Y-maze spontaneous alternation test, water maze, and passive avoidance tests, similar to the Normal-Con group. AD-F-L also potentiated hippocampal insulin signaling and increased the expression of ciliary neurotrophic factor (CNTF) and brain-derived neurotrophic factor (BDNF) in the hippocampus. AD-C-L improved the memory function mainly by increasing the BDNF content. AD-F-H and AD-C-H did not improve the memory function. Compared to AD-Con, the ascending order of AD-C-H, AD-F-H, AD-C-L, and AD-F-L increased insulin signaling by enhancing the pSTAT3→pAkt→pGSK-3β pathway. AD-F-L improved glucose tolerance the most. Compared to AD-CON, the AD-F-L treatment increased the serum acetate concentrations and compensated for the defect of cerebral glucose metabolism. AD-Con increased *Clostridium*, *Terrisporobacter* and *Sporofaciens* compared to Normal-Con, and AD-F-L and AD-C-L increased *Akkermentia*. In conclusion, AD-F-L and AD-C-L alleviated the memory function in the rats with induced AD symptoms by modulating.

## 1. Introduction

Alzheimer’s disease (AD) is the most common type of dementia, affecting people over 65 years of age [1]. AD is a complex neurodegenerative disease that shows cognitive dysfunctions involving attention, comprehension, memory, judgment, and language, which interfere with daily life [2]. Diabetes, hypertension, obesity, and dyslipidemia are risk factors for cognitive decline [3]. Although the increased inflammation associated with these diseases interacts with dementia through complex mechanisms, the relationship between increased inflammation and the perturbed insulin/insulin-like growth factor (IGF)-1 signaling pathway in the brain is consistent [3]. Previous studies have shown that a disrupted insulin signaling pathway contributes to the neurodegenerative process in patients with hyperglycemia, hyperlipidemia, and vascular disease [4]. Multiple studies have confirmed that improving brain insulin signaling is essential for dementia [5]. AD-related pathologies mainly include amyloid-β (Aβ) accumulation, neurofibrillary tangles, and neuronal necrosis [6]. Many hypotheses on the AD etiology have been proposed based on these pathological features, including the Aβ theory, the tau protein theory, genetic mutations, synaptic damage, inflammation, or mitochondrial dysfunction [6,7]. Conversely, none of these hypotheses can fully explain AD pathogenesis [7].

Dietary fiber is an essential nutrient for maintaining the normal physiological functions of the human body [8]. Dietary fiber increases satiety, reduces food intake, and promotes intestinal movement. In addition, dietary fiber regulates the intestinal flora community to enhance the α-diversity and beneficial bacteria and resist the development of various diseases in the host [8]. In particular, soluble dietary fiber helps reduce metabolic disorders, including Alzheimer’s disease, by modulating the gut bacteria community. On the other hand, different dietary fiber sources and properties contribute to the diverse effects on AD [8]. Soluble dietary fiber is easily metabolized by specific gut microbiota to produce functional metabolites such as short-chain fatty acids (SCFAs), essential for regulating the host metabolism [9,10]. They have various functions in energy metabolism, anti-inflammatory, insulin sensitivity, and neuronal function [11,12]. Propionate and butyrate among SCFAs improve brain insulin sensitivity through the gut-brain axis [13] and enhance parasympathetic dysfunction between the brain and the intestines. They contribute to cognitive function [14]. Dietary fiber also modulates the gut microbiota community to change bile acid reabsorption and metabolism, potentially influencing the host metabolism and brain function through the gut-brain axis [14,15].

Over the past few decades, natural products have played a vital role in drug discovery and development. Various natural products, mainly from plant sources (roots, bark, flowers, or essential oils) and algae-derived compounds, are considered effective alternatives to treat multiple diseases [16,17,18]. Seaweeds and algae contain a rich and valuable source of bioactive compounds to develop natural products and prevent or treat various diseases for many years. They are still being used in healthcare in many countries [19,20]. Marine sulfated polysaccharides, including fucoidans and carrageenan [20], show various pharmacological activities, such as antitumor, antiviral, antioxidant, antibacterial, anticoagulant, and immunoinflammatory effects, by modulating the gut microbiota and their metabolites [20]. They demonstrate the improvement of cognitive function and the suppression of neuronal damage by reducing oxidative stress and inflammation in Aβ(1–40 or 1–42) injected rats and cell-based studies [21,22,23]. They are a potential source for drug development that alleviates memory function [21,22,23]. Microbial accessibility to dietary fiber may promote beneficial activity to prevent diseases [24]. Dietary fiber with different molecular weights (MW) may have other activities in alleviating AD symptoms by modulating the gut microbiota community and SCFAs. The fermentation of fucoidan and λ-carrageenan generates smaller MW products. The efficacy of unfermented and fermented fucoidan and λ-carrageenan on cognitive function accounts for the microbial accessibility in the gut. The present study hypothesized that the low-MW and high-MW of fucoidan and λ-carrageenan would synergistically improve memory impairment in rats with AD induced by an infusion of toxic Aβ. We also explored its action mechanism.

## 2. Materials and Methods

### 2.1. Fermentation and Freeze-Drying of Fucoidan and λ-Carrageenan

*Luteolibacter algae* (KCTC 22040) and *Pseudoalteromonas carrageenovora* (KCTC 22325) were reported to make low-MW fucoidan and λ-carrageenan, respectively [25]. They were purchased from Korean Collection for Type Cultures to make low-MW fucoidan and λ-carrageenan. According to the method guidelines, the strains were activated and stored at −70 °C in a preservation solution containing 20% glycerol. After activating *luteolibacter algae* and *Pseudoalteromonas carrageenovora* for 12 h before inoculation, they were added to 1% fucoidan and λ-carrageenan in Difco marine broth for fermentation, respectively [26,27]. The initial inoculation concentration was 2 × 10^5^ colony-forming units (CFU)/mL, followed by incubation at 180 rpm and 37 °C for 48 h. After fermentation, the broth was sterilized by centrifugation (6000 rpm/min) to kill the bacteria. The final ethanol concentration of the culture broth reached 60% by adding ethanol. After refrigeration overnight, the protein was removed by centrifugation at 3000 rpm/min. The supernatant was freeze-dried to obtain the algal polysaccharides.

Before and after fermentation of fucoidan and λ-carrageenan, their amounts were analyzed according to the molecular size by gel permeation chromatography/preparative gel permeation chromatography (GPC/PGP; Omnisec, Malvern, UK). The machine was equipped with an ultrahydrogel column with 250, 500, 1000, and 2000 sizes (7.8 mm × 300 mm), eluted with water, 1 mL/min flow rate, and a refractive index detector.

### 2.2. Animal Care and Cannulation Surgery into the Bilateral CA1 Subregions of the Hippocampus

Male Sprague-Dawley rats, weighing 267 ± 15 g, were purchased from Dae Han Bio Link Inc. (DBL; Um-Sung, Korea). They were housed individually in stainless steel cages and allowed one week to acclimate. The ambient temperature and humidity were 25 ± 5 °C at 65 ± 5%, respectively, with a 12-h light (08:00–20:00) and dark (20:00–08:00) cycle. The rats were given free access to rod-feed (DBL, Rod feed, Korea) and water during the acclimation period. All experimental research procedures were conducted under the “Guidelines for the Care and Use of Laboratory Animals” of the national institutes of health and were approved by the animal care and use review committee of Invivo company (Nonsan, Korea (IV-RB-02-2020-11).

The rats were given the assigned diet for 20 days, and surgery to infuse Aβ(25–35) was performed on the 21st day. After being anesthetized with an intraperitoneal injection of ketamine and xylazine (100 and 10 mg/kg body weight, respectively; Bayer CropScience, Leverkusen, Germany), the rats were placed in a stereotaxic apparatus. A stainless-steel cannula was implanted in the bilateral CA1 subregions of the hippocampus according to the coordinates (lateral, −3.3 mm from the bregma; posterior, 2.0 mm from the midline; ventral, −2.5 mm from dura). The Aβ(25–35) peptide was reported to self-associate like the full Aβ(1–42) peptide by switching to insoluble β structures [28]. The Aβ(25–35) peptides eventually make fibrils much faster than the longer Aβ peptide [28], and its injection Aβ accumulation and neuronal morphological changes in the hippocampus [29]. It induces a neurotoxic effect through cholesterol-dependent membrane pore formation in the brain and neuroinflammation to exacerbate Alzheimer’s disease-like symptoms [30]. Aβ(25–35) for each rat was diluted to 0.005 mg in 300 µL with sterile distilled water, and it was filled in an osmotic pump (Alzet Osmotic Pump Company, Cupertino, CA, USA). The implanted canular was connected to the Aβ-filled osmotic pump (Alzet Osmotic Pump Company, Cupertino, CA, USA) to connect to the cannula implanted in the bilateral CA1 subregions. The Aβ(25–35) was infused continuously at 0.5 µL per hour for three weeks.

### 2.3. Experimental Design and Diets

The freeze-dried low- and high-MW fucoidan and λ-carrageenan were ground to a powder. Based on previous studies, the required amount was estimated for the intervention. Dyslipidemia and atherosclerotic lesion were inhibited in hyperlipidemic mice fed a high-fat diet containing 1% and 5% fucoidan for 12 weeks in a dose-dependent manner [31]. The present study examined whether the low-MW fucoidan and λ-carrageenan had better efficacy in memory impairment than high-MW alternatives; 1% low- and high-MW fucoidan and λ-carrageenan were added into a high-fat diet according to the assigned group. Dextrin (1%) instead of fucoidan and λ-carrageenan was supplemented into the high-fat diet. All rats had a high-fat diet to exacerbate memory dysfunction by elevating brain insulin resistance and neuroinflammation in Aβ(25–35)-infused rats [32,33,34]. The high-fat diets were made based on a modified American Institute of Nutrition-93 diet, and 1% dextrin, fucoidan, or λ-carrageenan were substituted for cellulose [35]. The primary dietary sources of carbohydrate, protein, and fat were starch plus sugar, casein, and lard plus corn oil (CJ Co., Seoul, Korea), respectively. The diets consisted of approximately 42 energy percent (En%) carbohydrates, 15 En% protein, and 43 En% fats.

**Figure 1:**
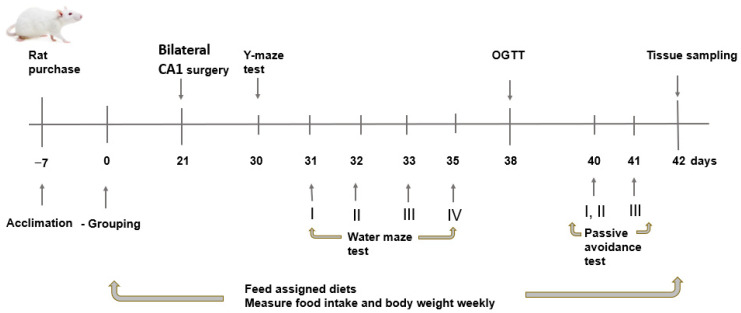
Experimental design. After grouping the rats, they were fed a high-fat diet containing dextrin (AD-Con), fucoidan with high molecular weight (MW; AD-F-H), fucoidan with low-MW (AD-F-L), λ-carrageenan with high MW (AD-C-H), or λ-carrageenan with low MW weight (AD-C-L). The rats in the Normal-Con had hippocampal saline infusion to have no amyloid-β deposition and were fed a high-fat diet (Normal-Con). At 21 days from feeding the assigned diet, a stainless-steel cannula was implanted in the bilateral CA1 subregions of the hippocampus, and Aβ(25–35) fragment was infused into the hippocampus for 28 days. The assigned diet was provided until euthanizing of the rats. OGTT, oral glucose tolerance test.

Fifty Aβ(25–35) infused (AD) rats were divided into five groups with ten rats in each group: (1) 1% dextrin (AD-Con), (2) 1% fucoidan high-MW (AD-F-H), (3) 1% fucoidan low-MW (AD-F-L), (4) 1% λ-carrageenan high-MW (AD-C-H), and (5) 1% λ-carrageenan low-MW (AD-C-L). Ten rats were infused with saline instead of Aβ(25–35) and were provided a 1% dextrin-containing diet (Normal-Con). All rats were given free access to water and food. The experiment was conducted for 42 days; Figure 1 presents the experimental scheme.

### 2.4. Y-Maze Spontaneous Alternation Test

On the 30th day of the experiment, a Y-maze test was conducted to assess the cognitive function of the rats. A Y-shaped plexiglass cage contained three channels with an angle of 120°, and each channel was 50.5 cm long, 20 cm wide, and 20 cm high. Each rat started walking from channel A, and its walking route was recorded for 8 min. The route to travel in different directions (A-B-C, B-C-A, and C-A-B) and repetition of the same direction (A-B-A, B-A-B, and C-A-C) were scored into 1 and 0 points, respectively. The alteration percentage was calculated as follow: alternation % = [(number of right alternation)/(total number of arm entries − 2)] × 100. After experimenting with a single rat, the dirty sawdust in all passages was replaced with a new one to prevent odor.

### 2.5. Morris Water Maze Test

From the 31–35th day of the experiment, the spatial memory function of the rats was assessed using the Morris water maze test. The water maze test was carried out in a circular pool with a diameter of 180 cm and a height of 50 cm. The swimming pool was filled with water to a depth of 35 cm and a temperature of 23 ± 1 °C and placed in black edible dye to make the water black to see the rat better. The pool was divided into five zones, and a fixed spot, called zone 5, was chosen where a circular platform (a diameter of 10 cm and a height of 30 cm) was placed. The water depth was more than 2 cm above the platform. A water maze test consisted of two training trials with a platform in a fixed position on a consecutive day. The rat was placed individually in the pool facing the wall to eliminate any directional disturbance at the starting point. When the rat swam to the pool for eight minutes, a trained researcher observed a latency time to climb on the platform. If the rat failed to find the podium during the trial period, the researcher guided the rat to the platform and remained there for 30 s, which was the learning process. On the fourth day, the rat found the area where the podium was in the pool. After each animal experiment, the animal was dried entirely with a dry paper towel and warm wind before returning to the rearing room. A video camera recorded the free movement of the rat and measured the distance and time spent to enter the zone where the platform was located on the third trial.

### 2.6. Passive Avoidance Test

On days 40 and 41, a passive avoidance test was conducted [36] to measure short-term memory. The short-term memory deficit in rats was estimated using a passive avoidance apparatus consisting of a two-chamber (lighted room/dark room) shuttle box [36]. On the first day, after the rat was located in room A (lighted room), it remained in room A for five minutes. In the loud condition, room B (dark room) was opened simultaneously, and the rat’s foot entered room B and was immediately given a small electrical stimulus (75 V, 0.3 mA, 3 s). After the first trial, the rat was removed from the dark room and returned to its cage. The rat had a two-hour rest. A second memory test was performed to determine if it had learned not to enter the darkroom. The next day, the rat had the passive avoidance test without electrical stimulation. Retention latency to enter the darkroom was recorded for 360 s in each trial. Shorter latencies indicated poorer memory in rats compared to significantly longer latencies.

### 2.7. Serum Glucose-Insulin Levels during Oral Glucose Tolerance

On the 29th day, the fasted rats were given an oral intake of 2 g glucose/kg body weight, called the oral glucose tolerance test (OGTT). During the OGTT, the glucose concentrations of the tail blood were measured every 10 min until 90 min, and then again at 120 min using a blood glucose meter (Accucheck; Roche Diagnostics, Basel, Switzerland). Blood from the tail was drawn at 0, 20, 40, and 90 min to measure the serum insulin concentrations.

### 2.8. Sample Collection and Biochemical Assays

After 42 days of treatment, the rats were anesthetized with ketamine and xylazine (100 and 10 mg/kg body weight, respectively). Venous blood was collected from the vena cava, and the serum was separated by centrifugation at 500 rpm for 4 min. The liver and epididymal fat mass were weighed and quickly snap-frozen in liquid nitrogen. After collecting the brain, the hippocampus portion of five rats in each group was separated and stored in a −70 °C freezer. The remaining five brains were stored in the 20% sucrose solution for two days and then frozen in a −20 °C freezer. Feces were collected from the rat’s cecum, and the liver and feces were stored in a −70 °C freezer.

According to the kit instructions, serum aspartate aminotransferase (AST), alanine aminotransferase (ALT), total cholesterol (TC), and high-density lipoprotein cholesterol (HDL-C) concentrations were detected using colorimetric kits (Asan Pharmaceutical Co., Seoul, Korea). The liver and brain tissue were lysed with ultrasound in a methanol solution (1:10) and mixed with a chloroform solution. After adding distilled water, the upper layer containing the organic solvent was separated after centrifuging the mixture at 3000 rpm for 10 min at 4 °C. The upper layer was mixed with ethanol plus triton X mixture (1:1, *v*/*v*). The triglyceride (TG) and TC contents in the emulsified upper layer were measured using TG and TC kits (Asan Pharmaceutical Co., Seoul, Korea). The liver and brain tissues were lysed in a 0.3 N perchloric acid solution by ultrasonication, and then the lysates were mixed with a 1 N NaHCO_3_ solution and an α-amyloglucosidase solution in sodium acetate. The mixture was incubated in a 37 °C incubator for 18 h. According to the instructions, the glucose concentrations in the supernatants separated by centrifugation were measured by a glucose kit (Asan Pharmaceutical, Daejeon, Korea). Glycogen contents were calculated in the liver and brain tissue.

### 2.9. Brain Immunohistochemistry

Cryoprotected brain frozen tissues stored in 20% sucrose solution were sectioned serially on a cryostat (Leica, Wetzlar, Germany) into 30 μm coronal sections, and Aβ deposition was determined using the Aβ antibody by immunohistochemistry [36]. The Aβ deposition was calculated from the % Aβ^+^ cells in the hippocampus.

### 2.10. MRNA Expression in the Brain by Real-Time Quantitative Reverse Transcription-Polymerase Chain Reaction (RT-PCR)

Hippocampal tissue (*n* = 5) was lysed with Trizol (Ambion Inc., Austin, TX, USA) reagent solution, and the total RNA was isolated. The amount and purity of the total RNA were determined at 260 nm and 280 nm using a spectrometer (Perkin Elmer, Boston, MA, USA). According to the manufacturer’s instructions, the total RNA (1 µg) was synthesized into cDNA using the Superscript™ III Reverse Transcriptase Kit (Bio-Rad, Richmond, CA, USA). The synthesized cDNA was mixed with SYBR Green supermix (Bio-Rad, Richmond, CA, USA) and amplified using the CFX Connect™ Real-Time PCR Detection System (Bio-Rad Laboratories, Inc., Hercules, CA, USA). The relative mRNA expression of the ciliary neurotrophic factor (CNTF) and brain-derived neurotrophic factor (BDNF) in the hippocampus was assessed using the cycle of threshold (CT) method. The gene expression levels were normalized with those of the housekeeping gene β-actin. The primers for the gene were as follows: β-actin: Forward: 5′-AGCGTGGCTACAGCTTCACC-3′, Reverse: 5′-AAGTCTAGGGCAACATAGCACAGC-3′. BDNF: Forward: 5′-ATGCCGAACTACCCAATCGT-3′, Reverse: 5′-GCCAATTCTCTTTTTGCTATCCA-3′. CNTF: Forward: 5′-ATGGCTTTCGCAGAGCAATCACC-3′, Reverse; 5′-GTCGTGTACTCTCGGTAATACC-3′.

### 2.11. Western Blot Analysis

The hippocampus (*n* = 4) was lysed with a radioimmunoprecipitation assay (RIPA) buffer supplemented with protease inhibitors. The amount of protein in the supernatants was measured using a Bio-Rad protein assay kit (Bio-Rad, Hercules, CA, USA). Western blot analysis was performed, as described previously [37]. Briefly, the brain tissue was centrifuged at 14,000 rpm for 20 min in a solution containing protease and phosphatase inhibitors (Pierce Biotechnology, Rockford, IL, USA), and the supernatant was collected. The proteins in the supernatants (50 μg protein) were loaded, separated using sodium dodecyl sulfate-polyacrylamide gel electrophoresis, and then transferred to nitrocellulose membranes. We chose some proteins involved in improving glucose metabolism through the insulin signaling pathway to modulate hippocampal memory function, as shown in previous studies [35,38,39]. The selected primary antibodies, e β-actin, Akt, phosphorylated Akt, signal transducer and activator of transcription-3 (STAT3), phosphorylated STAT3, glycogen-synthase kinase (GSK)-3β, and phosphorylated GSK-3β antibodies (1:1000; Chemicon, Temecula, CA, USA) were reacted with the transferred membranes at 4 °C overnight. The membranes were then incubated with Alexa Fluor 700-conjugated anti-rabbit IgG-HRP (1:20000; Invitrogen, Eugene, OR, USA) for 30 min. The specific bands for the genes of interest were detected using ECL kits, and their intensity was measured by optical densitometry (I-Solution software, IMT *i*-solution Inc., Burnaby, BC, Canada).

### 2.12. Determination of the Serum SCFA Concentrations

SCFAs, including acetate, propionate, and butyrate, were produced in the large intestines from the specific gut microbes by fermenting indigestible dietary fibers [40]. They entered the circulation through the portal vein. The serum samples from the portal vein were filtered using a 0.45 µm microporous membrane filter and analyzed for SCFA concentrations using a Clarus 680 gas chromatograph (PerkinElmer, Waltham, MA, USA) containing a column (Elite-FFAP 30 m × 0.25 mm) [41]. The injection volume was 1 µL. The initial temperature was 100 °C, and it was then raised to 180 °C at 10 °C/min, increased to 220 °C at 20 °C/min, and held at that temperature for eight minutes. The inlet and flame ionization detector temperatures were 220 °C and 240 °C, respectively. The helium, nitrogen, and hydrogen flow rates were 20 mL/min, 450 mL/min, and 45 mL/min, respectively. The standards for butyric, propionic, and acetic acid (Sigma Co., St. Louise, MO, USA) were 5, 2, and 1 mM, respectively.

### 2.13. Next-Generation Sequencing (NGS) of Gut Microbes

The total genomic DNA of fecal bacteria in the cecum was extracted using a QIAamp Power Fecal DNA Kit (QIAGEN, Düsseldorf, Germany). The gut microbiome from the DNA was measured using NGS, a high-throughput DNA sequencing technology. The extracted DNA samples were normalized to 5 ng/µL using diethyl pyrocarbonate water (Sigma Co., St. Louise, MO, USA). The primers of the V3–V4 rRNA region contained sufficient genetic information on the bacteria characteristics and were used to amplify the isolated DNA [42]. The extracted bacteria DNA, primers, and polymerase with the proper buffers in the KAPA HiFi HotStart Ready Mix PCR Kit (KAPA Biosystems, Wilmington, MA USA) were mixed. Amplification was conducted using GeneAmp PCR under the following conditions: 94 °C for three minutes, followed by 35 cycles: 94 °C for 15 s, 55 °C for 45 s, followed by one minute at 72 °C, and by eight minutes of extension at 72 °C. The polymerase chain reaction (PCR) amplicons were visualized on agarose gels using a QIAquick PCR purification kit (Qiagen, Valencia, CA, USA), and their concentrations were measured with Nanodrop 2000 (Thermo Fisher Scientific, Waltham, MA, USA). The amplified PCR products were purified using AMPure beads (Beckman Coulter, Brea, CA, USA), and the purified samples were sent to Macrogen Ltd. (Seoul, Korea) for sequencing using the Next generation sequencing (NGS) method.

The sequencing data from NGS were analyzed using Mothur (version 1.43.0). Bidirectional sequences were first merged, and sequences > 200 bp were filtered out. Clustering was performed, and the similarity threshold was 97%. The identified chimera was removed using the chimera vsearch command (identification threshold = 3). We annotated the processed sequence files according to the Greengenes reference taxonomy. The α-diversity by the Shannon index was calculated using a single summary tool with the count tables and taxonomy files. The β-diversity was determined using an unweighted unifrac tool, and Lefse analysis was performed using the Lefse command.

### 2.14. Picrust2 Analysis

Functional abundance analysis of the gene sequences was performed with the processed fasta files and count files using Picrust2 (version 2.3.0_b). After converting the count file from the Mothur process into biom format, the sequence files and biom file were introduced into Picrust2 to run for metagenome analysis. The Kyoto Encyclopedia of Genes and Genomes orthology (KO) abundance was modified to the relative abundance, and then the website (https://www.genome.jp/kegg/tool/map_pathway1.html, accessed on 5 January 2022) was used to obtain 341 related metabolic pathways.

### 2.15. Statistical Analysis

Results were analyzed using SPSS 16.0 (IBM, Chicago, IL, USA). Data shown are expressed as mean ± standard deviations. The data were analyzed using a one-way analysis of variance (ANOVA) followed by post hoc comparisons between the groups using the Tukey test. One-way repeated-measure ANOVA (treatment × time) was applied to OGTT results. The sequential data from the training and experimental trials in the water maze and passive avoidance tests were statistically analyzed with two-way repeated measured ANOVA. The statistical significance was set at *p* < 0.05.

## 3. Results

### 3.1. Amounts of Different MW before and after Fucoidan and λ-Carrageenan Fermentation

Before fermentation, fucoidan contained 100% of 88.959 kilodaltons (kD), and λ-carrageenan comprised 100% of 974.6 kD (Supplementary Figure S1). After fermentation, fucoidan was degraded to 34.2% of 13.915 kD, 43.7% of 2.260 kD, and 22.14% of 178 daltons (D), while λ-carrageenan was broken down into 6.11% of 2.672 kD, 13.7% of 1.319 kD and 456 D, and 1.83% of 185 D. The GPC/GFC analysis results showed that fucoidan and λ-carrageenan were degraded into smaller molecules.

### 3.2. Energy Metabolism

The final body weight and weight gain during the intervention were lower in the AD-Con group than in the Norma-Con group (*p* < 0.05), and AD-F-L and AD-C-L increased it to as much as in the Normal-Con group (Table 1). The food efficiency was lower in the AD-Con than in the Normal-Con group, and in the AD-F-L and AD-C-L groups this decrease was inhibited (*p* < 0.05). On the other hand, the visceral fat mass, a sum of the epididymal and retroperitoneal fat mass, was not significantly different among the groups (Table 1).

### 3.3. Aβ Deposition in the Hippocampus and Y-Maze Test

**Figure 2:**
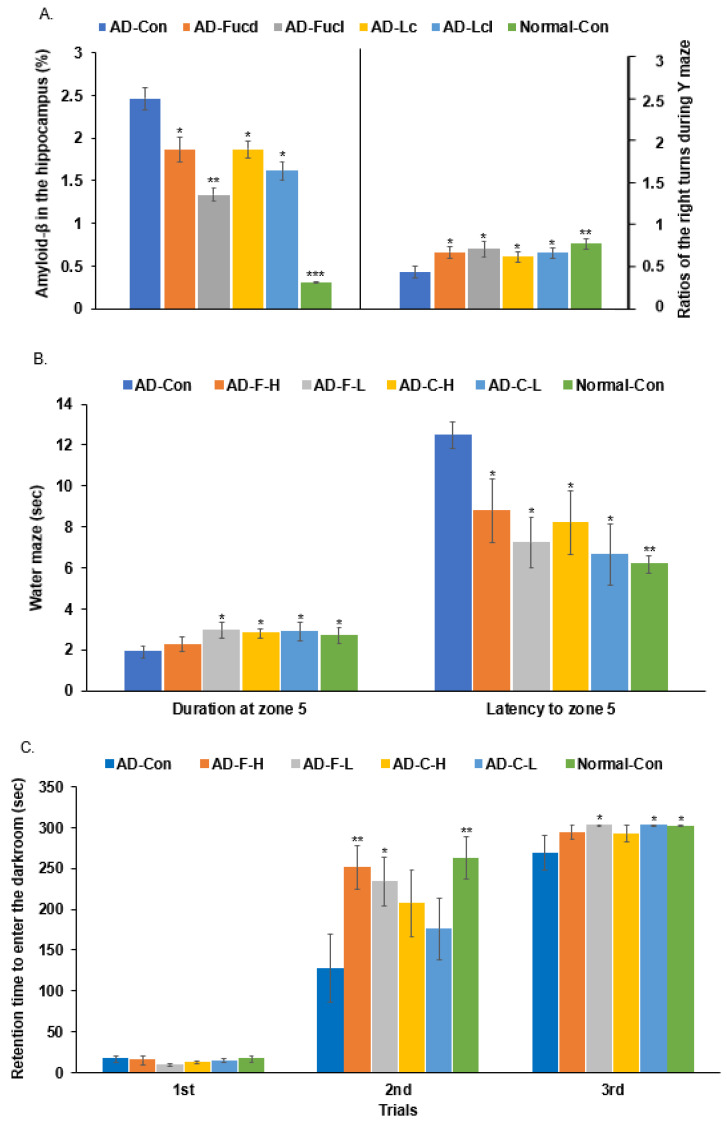
Memory deficits in rats after hippocampal amyloid-β infusion. (**A**) Percentage of correct alternations to total rotations in the Y maze test. (**B**) The frequencies of visiting zone 5 where the platform was located, time spent in zone 5, and time spent finding the target area (zone 5) in the third trial in the water maze test. (**C**) Latency time to enter the darkened room in the passive avoidance test in two training trials every 8 h and the last trial 24 h later. The bars and error bars represent the means ± standard deviations (*n* = 10). Hippocampal amyloid-β(25–35) infused rats were fed a high-fat diet containing dextrin (AD-Con), fucoidan with high molecular weight (MW; AD-F-H), fucoidan with low-MW (AD-F-L), λ-carrageenan with high molecular weight (AD-C-H), and λ-carrageenan with low molecular weight (AD-C-L). The rats in the Normal-Con group had hippocampal saline infusion to have no amyloid-β deposition and were fed a high-fat diet (Normal-Con). * Significant from the AD-Con group at *p* < 0.05, ** at *p* < 0.01, and *** at *p* < 0.001.

Approximately 2.4% Aβ deposition was detected in the hippocampus of the AD-con group. The AD-F-H, AD-C-H, and AD-C-L groups had lower Aβ deposition than the AD-Con group, whereas the AD-F-L group had the least deposition among the groups (Figure 2A). However, the Aβ deposition in the AD-F and AD-L groups was much higher than in the Normal-Con group, suggesting the fucoidan and carrageenan could not inhibit Aβ deposition entirely.

The cognitive function tests involved counting the total number of turns in the arm of the Y maze. When the rats turned in a single direction in the Y maze, it was considered the correct turn, and returning to the previous passage was counted as the wrong turn. Compared to the Normal-Con group, the ratio of right turns to the total moves in the AD-Con group was lower than that in the Normal-Con group. The proportions of the AD-F-H, AD-F-L, and AD-C-L groups were higher than those of the AD-Con group and similar to those of the Normal-Con group (Figure 2A).

### 3.4. Morris Water Maze and Passive Avoidance Tests

After two training sessions of the water maze test, the rats in the AD-F-L, AD-C-L, and AD-C-H groups arrived quickly in zone 5, where the platform had been placed, compared to the AD-Con, and they showed a similar latency to zone 5 as the Normal-Con group in the third trial of water maze test. The AD-F-L and AD-C-L groups stayed longer in zone 5 to find the platform than those in the AD-Con group (Figure 2B). The result suggested that the rats in the AD-F-L and AD-C-L groups exhibited the most remarkable improvement in spatial memory function.

In the first passive avoidance test, all rats entered a dark room and received small electric shocks on the soles of their feet. Compared to the Normal-Con group, there was no difference between the groups (Figure 2C). When the second passive avoidance experiment was performed two hours later, the rats in the AD-Con group had a shorter latency period, indicating that the rats in the AD-Con group forgot the training session. In contrast, the AD-F-H and AD-F-L groups delayed entering the dark room, similar to the Normal-Con group (Figure 2C). After 18 h, in the third trial, the AD-F-L, AD-C-L, and AD-F-L rats did not enter the dark room, similar to the Normal-Con group (Figure 2C). Therefore, in the first, second, and third trials, the AD-F-L group showed enhanced short-term memory, which was improved partially by AD-C-L.

### 3.5. Glucose Metabolism by OGTT

**Figure 3:**
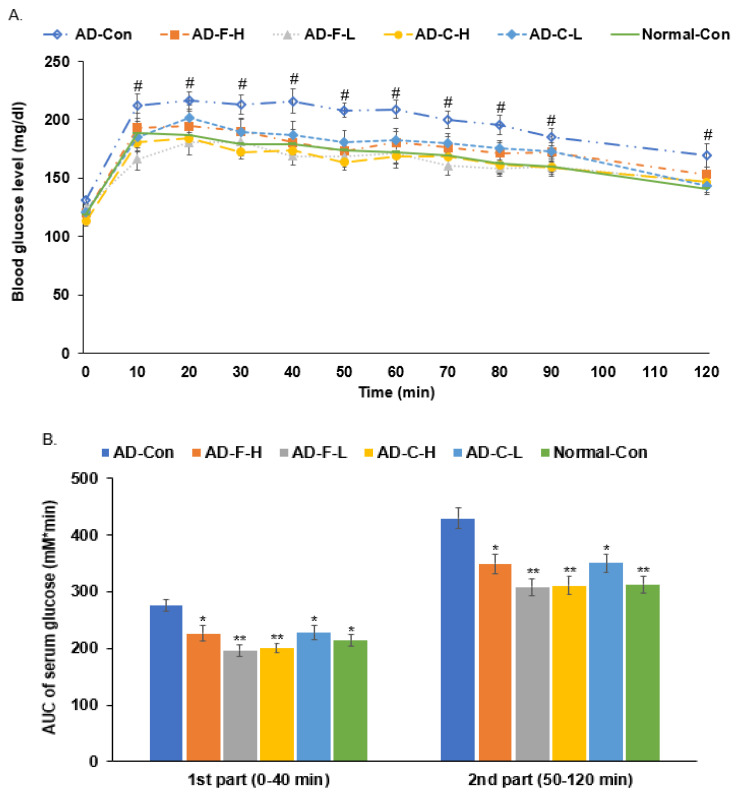
Serum glucose concentrations during oral glucose tolerance test. (**A**) Serum glucose levels at an assigned time after the glucose challenge (2 g/kg body weight). Treatment effect at *p* < 0.01, time effect at *p* < 0.01, and interaction between treatments and time at *p* < 0.01 on serum glucose concentrations by one-way repeated ANOVA. ^#^ Significant treatment effects at *p* < 0.05. (**B**) The area under the curve (AUC) of glucose in the first (0–40 min) and second phases (50–120 min). The dots or bars and error bars represent the means ± standard deviations (*n* = 10). Hippocampal amyloid-β(25–35) infused rats were fed a high-fat diet containing dextrin (AD-Con), fucoidan with high molecular weight (AD-F-H), fucoidan with low molecular weight (AD-F-L), λ-carrageenan with high molecular weight (AD-C-H), and λ-carrageenan with low molecular weight (AD-C-L). The rats in the Normal-Con group had hippocampal saline infusion to have no amyloid-β deposition and were fed a high-fat diet (Normal-Con). * Significant from the AD-Con group at *p* < 0.05 and ** at *p* < 0.01.

Fasting blood glucose concentrations were similar in all groups. After the glucose load, blood glucose concentrations increased in all groups. One-way repeated ANOVA exhibited a significant main effect of the treatments (*p* < 0.01), time (*p* < 0.01), and interaction between treatments and time (*p* < 0.01) on serum glucose concentrations during OGTT. Serum glucose concentrations were significantly higher in the AD-Con group at 10, 40, 50, 70, 80, and 120 min, and lowest in the AD-F-L group than in other groups at each time point by one-way ANOVA (*p* < 0.05). At 50 min, the blood glucose of the AD-C-H group also decreased compared to the AD-Con group. The blood glucose concentration of the AD-Con group increased from 0–40 min to peak, started to decline slowly from 50 min, continued to 120 min, still higher than that of the Normal-Con, AD-F-L, AD-C-L, and AD-C-H groups (*p* < 0.05) (Figure 3A). In addition, the blood glucose levels of the Normal-Con, AD-F-L, AD-F-H, AD-C-L, and AD-C-H groups all decreased after reaching a peak. The blood glucose levels in the AD-F-L group were consistently lower than those in the AD-F-H, AD-C-L, and AD-C-H groups (Figure 3A).

### 3.6. Liver Damage and Cholesterol Metabolism

The serum AST and ALT concentrations were not different among all groups, indicating that F-H, F-L, C-H, and C-L interventions did not induce liver damage in the AD rats. All groups showed similar levels of triglyceride accumulation in the liver and brain tissues (Table 2). On the other hand, the glycogen contents in the liver were higher in the Normal-Con group than in the AD-Con group, while AD-F-L and AD-C-L partly prevented the decrease (*p* < 0.05; Table 2). Like the liver glycogen contents, the glycogen storage in the brain tissue was higher in the Normal-Con group than in the AD-Con group. It increased in AD-C-L, AD-C-H, AD-F-L, and AD-F-H groups compared to the AD-Con group but not as much as in the Normal-Con group (*p* < 0.05; Table 2). The cholesterol contents in the brain did not differ between the AD-Con and Normal-Con groups. Compared to the AD-Con group, the AD-F-H, AD-C-L, and AD-C-H groups showed lower cholesterol contents in the brain tissue (*p* < 0.05) (Table 2). 

### 3.7. MRNA Expression of Neurotrophic Factors in the Hippocampus

**Figure 4:**
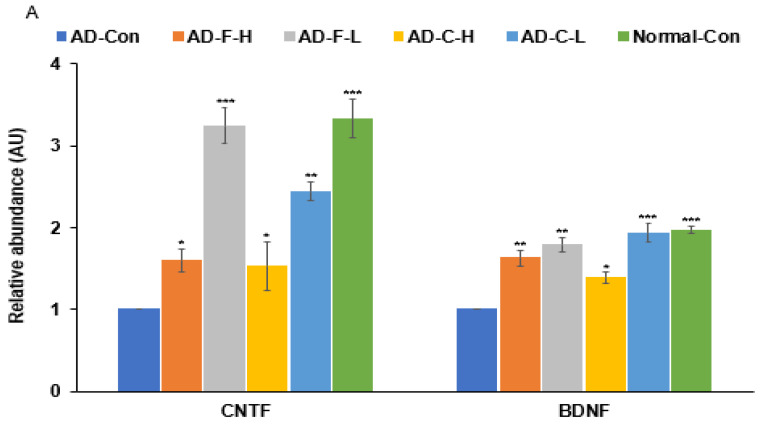

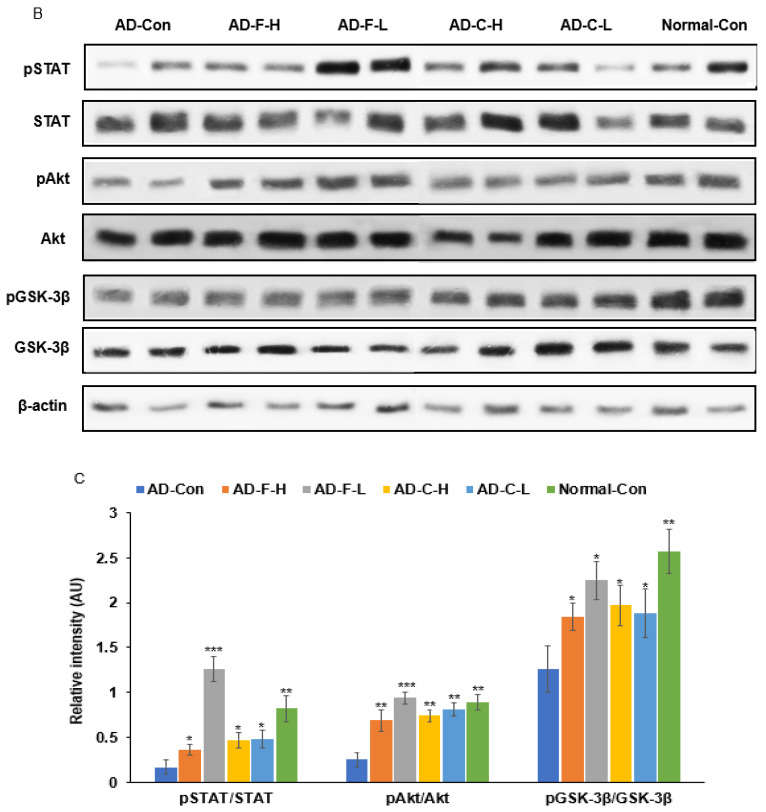
Hippocampal neurotransmitter expression and insulin signaling pathways. (**A**) mRNA expression of ciliary neurotrophic factor (CNTF) and brain-derived neurotrophic factor (BDNF). (**B**) Protein and phosphorylation of proteins in insulin signaling pathways. (**C**) The intensity of proteins and phosphorylation in insulin signaling pathways. The bars and error bars represent the means ± standard deviations (*n* = 4). Hippocampal amyloid-β(25–35) infused rats were fed a high-fat diet containing dextrin (AD-Con), fucoidan with high molecular weight (AD-F-H), fucoidan with low molecular weight (AD-F-L), λ-carrageenan with high molecular weight (AD-C-H), and λ-carrageenan with low molecular weight (AD-C-L). The rats in the Normal-Con group had hippocampal saline infusion to have no amyloid-β deposition and were fed a high-fat diet (Normal-Con). * Significant from the AD-Con group at *p* < 0.05, ** at *p* < 0.01, and *** at *p* < 0.001.

After treatment, the mRNA expressions of CNTF and BDNF in the brain tissue were much lower in the AD-Con group than in the Normal-Con group. The AD-F-H and AD-C-L groups showed similar CNTF mRNA expression to the AD-Con group, and AD-C-H showed similar BDNF mRNA expression to the AD-Con group. Compared to the AD-Con group, the AD-F-L group showed an increase in the mRNA expressions of CNTF and BDNF, similar to the Normal-Con group (*p* < 0.05). The AD-F-L and AD-C-L groups showed a similar increase in mRNA expression of BDNF to the Normal-Con group (*p* < 0.05) (Figure 4A).

### 3.8. Hippocampal Insulin Signaling

The phosphorylation of Akt, GSK-3β, and STAT3 interacts with insulin/IGF-1 signaling to prevent memory decline. The phosphorylation of the Akt, GSK-3β, and STAT3 proteins was significantly higher in the AD-F-L group than in the AD-Con group. The increase in Akt phosphorylation was higher in the AD-C-L group, but the increase was minimal in the other treatment groups (Figure 4B,C).

### 3.9. SCFA Concentrations in the Portal Vein Blood

The serum acetate concentration of the portal vein in AD-Con group was lower than in the Normal-Con group. Compared to the AD-Con group, the acetic acid concentration increased in the AD-F-L group, similar to that in the Normal-Con group (*p* < 0.05). The serum propionate concentrations were similar in all groups (Table 3). The serum butyric acid concentration was lower in the AD-Con group than in the Normal-Con group, but its decline was inhibited in the AD-F-L and AD-C-L groups (Table 3).

### 3.10. Gut Microbiota Community

**Figure 5:**
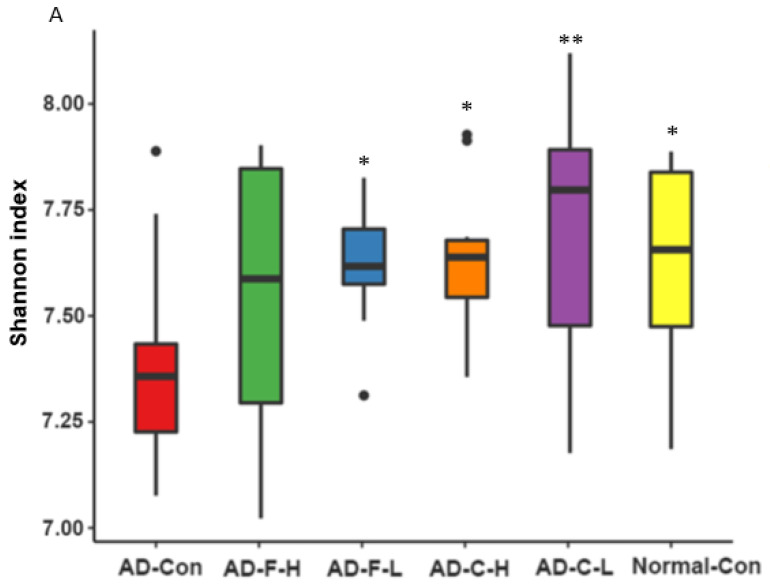

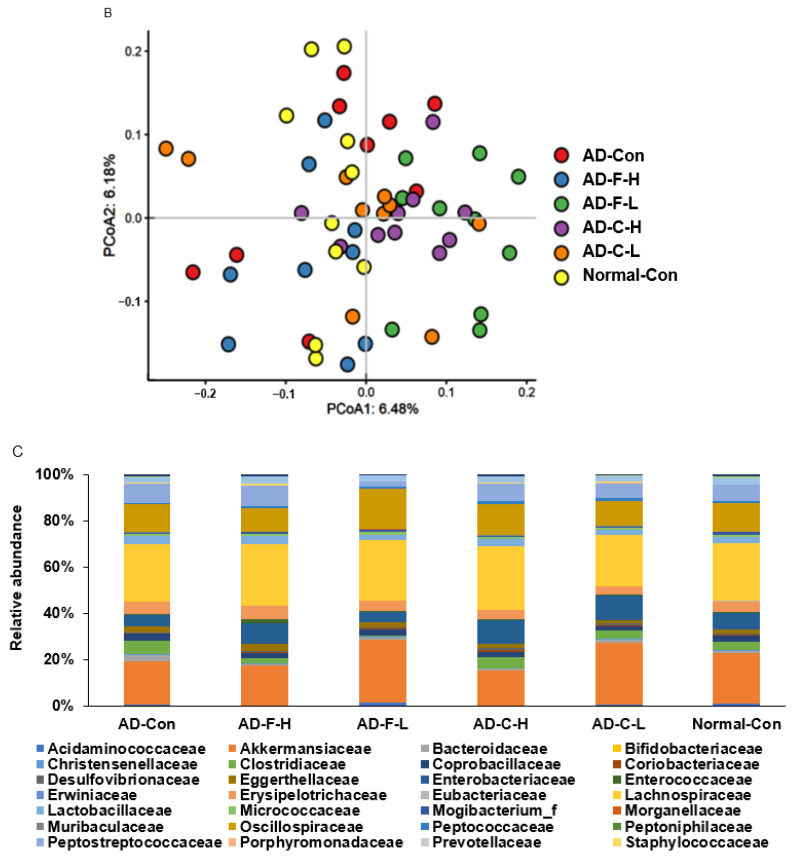

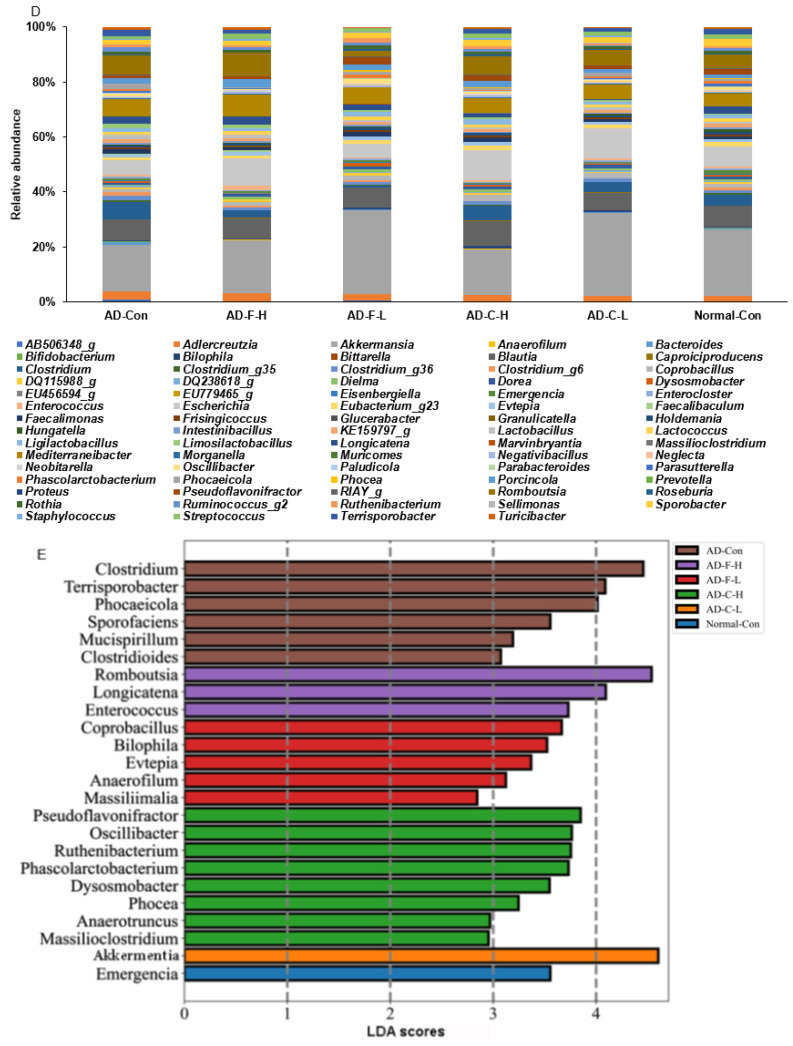
Gut microbiota community. (**A**) α-diversity. (**B**) β-diversity. (**C**) Relative abundance of cecum bacteria at the family level. (**D**) Relative abundance of cecum bacteria at the genus level. (**E**) LDA scores of specific bacteria among the groups in Lefse analysis. The bars and error bars represent the means ± standard deviations (*n* = 4). Hippocampal amyloid-β(25–35)-infused rats were fed a high-fat diet containing dextrin (AD-Con), fucoidan with high molecular weight (AD-F-H), fucoidan with low molecular weight (AD-F-L), λ-carrageenan with high molecular weight (AD-C-H), and λ-carrageenan with low molecular weight (AD-C-L). The rats in the Normal-Con group had hippocampal saline infusion to have no amyloid-β deposition and were fed a high-fat diet (Normal-Con). * Significant from the AD-Con group at *p* < 0.05 and ** at *p* < 0.01.

The α-diversity was lower in the AD-Con group than in the Normal-Con group, and its decrease was prevented in the AD-F-L and AD-C-H groups. AD-C-L increased the α-diversity compared to the Normal-Con group (Figure 5A). The β-diversity showed the separation between the AD-Con and Normal-Con groups, and AD-C and AD-F were separated from the AD-Con group (*p* < 0.05; Figure 5B).

At the family level, the AD-F-L and AD-C-L groups increased Akkermencease, whereas the AD-F-L elevated Oscillospiraceae and decreased Peptostreptococcaceae compared to the other groups (Figure 5C). The AD-F-L and AD-C-L groups showed elevated *Akkermentia* and *Escherichia* at the genus levels. The AD-F-L group exhibited lower *Romboutsia* and *Clostridium* levels than the other groups (Figure 5D). The AD-Con group had higher *Clostridium, Terrisporobacter*, *Phocaeicola*, *Sporofaciens*, *Mucispirillum*, and *Clostridiodes* levels than the other groups, and the Normal-Con group had higher *Emergencia* levels (Figure 5E). The AD-F-H group contained *Romboutsia, Longicatena*, and *Enterococcus*, while the AD-F-L group showed an increase in *Coprobacillus*, *Biophilia*, *Evtepia*, *Anaerofilum*, and *Massilimalia*. The AD-C-H group showed an elevation of *Pseudoflavonifractor*, *Oscillibacter*, *Ruthenibacterium*, *Phascolarctobacterium*, *Dysosmobacter*, *Phocea*, *Anerotruncus*, and *Massiloclostrodoum*, and the AD-C-L group had a higher level of *Akkermansia*. The AD-Con group included high potential pathogenic bacteria compared to the other groups. AD-F-L and AD-C-L did not produce an increase in pathogenic bacteria, while AD-C-L elevated some beneficial ones.

### 3.11. Metagenome Function of Gut Bacteria by Picrust2

**Figure 6:**
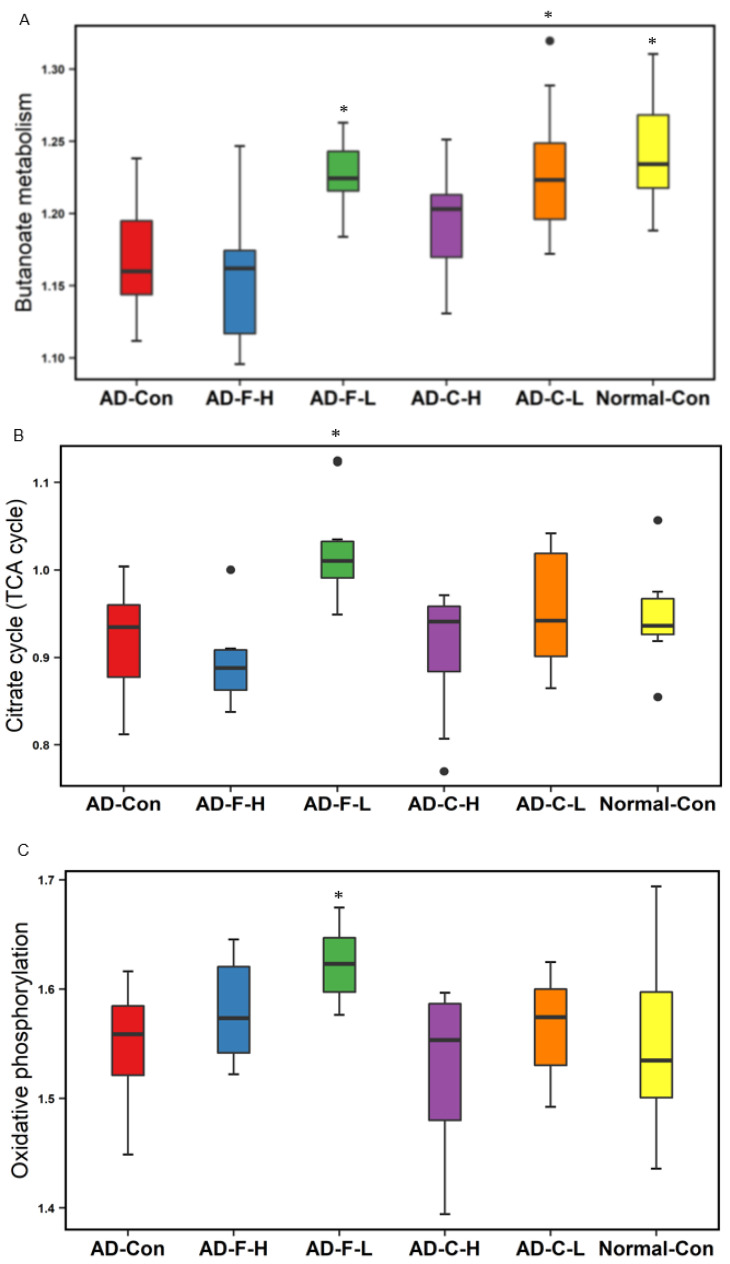

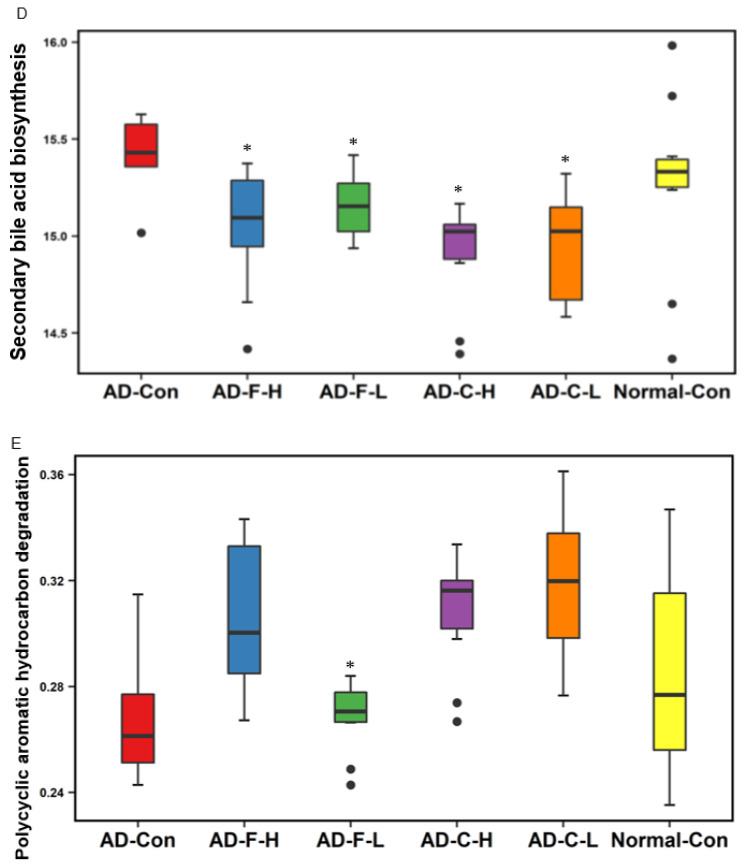
Metagenome function of cecum bacteria by picrust2 analysis. (**A**) Butanoate metabolism (**B**) Citrate cycle (TCA cycle, Krebs cycle) (**C**) Oxidative phosphorylation (**D**) Secondary bile acid biosynthesis (**E**) Polycyclic aromatic hydrocarbon degradation. Bars and error bars represent the means ± standard deviations (*n* = 10). Hippocampal amyloid-β(25–35)-infused rats were fed a high-fat diet containing dextrin (AD-Con), fucoidan with high molecular weight (AD-F-H), fucoidan with low molecular weight (AD-F-L), λ-carrageenan with high molecular weight (AD-C-H), and λ-carrageenan with low molecular weight (AD-C-L). The rats in the Normal-Con had hippocampal saline infusion to have no amyloid-β deposition and were fed a high-fat diet (Normal-Con). * Significant from the AD-Con group at *p* < 0.05.

Metagenome function analysis of fecal bacteria by Picrust2 showed that fucoidan and λ-carrageenan intervention modified the SCFAs, energy, secondary bile acid metabolism, and toxic compound degradation. Consistent with the serum butyric acid concentrations, they were lower in the Normal-Con group than in the AD-Con group, and the AD-F-L and AD-C-L groups showed an increase as much as the Normal-Con. The citric acid cycle and oxidative phosphorylation related to the energy metabolism were similar in the AD-Con and Normal-Con groups and elevated in the AD-F-L group compared to the other groups (Figure 6B,C). The biosynthesis of secondary metabolites, mainly from bile acids, was similar in the AD-Con and Normal-Con groups, but AD-F and AD-C intervention decreased the metabolism (Figure 6D). Interestingly, the AD-F-H, AD-C-H, and AD-C-L interventions increased the degradation of toxic compounds related to improving metabolic disorders, such as polycyclic aromatic hydrocarbons (Figure 6E).

## 4. Discussion

AD is the most common neurodegenerative disease in the elderly with complex etiologies, and its incidence is increasing due to longer life expectancy [1]. A growing literature confirms that chronic obesity, high cholesterol, high blood pressure, and diabetes are important factors inducing memory loss and impaired cognition [43]. Each of the risk factors increases the memory dysfunction risk by approximately twofold. In addition, combinations of risk factors also increase the overall risk of dementia by more than six times [3]. In particular, abnormal glucose metabolism is one of the characteristic changes of early AD, and subsequent AD development contributes to reduced glucose metabolism and energy production in the brain [44]. In the initial AD stage, glucose dysregulation in the brain accounts for the primary part of the metabolic activity of the brain. Interestingly, SCFAs are involved in glucose dysregulation in the brain [5]. Some studies suggested that SCFAs can be used as a substrate for the brain metabolism after being absorbed from the portal vein, and acetate, similar to ketone bodies, can pass through the blood−brain barrier, acting as an alternative energy source [2]. In addition, the brain can use butyrate and β-hydroxybutyrate as an energy substrate to generate energy, thereby compensating for the deficit of glucose metabolism in brain insulin-resistant states [45,46].

Furthermore, brain insulin resistance is closely related to systemic insulin resistance [39,47]. When the brain insulin resistance increases, the systemic insulin resistance is elevated and vice versa. Some studies showed that higher fasting insulin levels representing systemic insulin resistance might be associated with more significant declines in memory impairment. Bioactive components enhancing the glucose metabolism, such as luteolin, red pepper, and fermented soybeans, protect against memory dysfunction [13,48,49]. Although the effect of a high-fat diet alone remains controversial [33,50], it deteriorates both systemic and central insulin resistance and worsens hippocampal insulin signaling and cognitive dysfunction in animals with Alzheimer’s disease-like symptoms such as injecting Aβ(25–35) or Aβ(1–40 or 1–42) into the brain [34,51,52]. In the present study, a high-fat diet was fed to exacerbate cognitive dysfunction in AD-induced rats by infusing Aβ(25–35). Low- and high-MW fucoidan and λ-carrageenan supplementation in a high-fat diet improved glucose tolerance in AD-induced rats, and AD-F-L and AD-C-L interventions enhanced glucose tolerance the most. Consistent with the improvement of glucose tolerance, memory impairment was alleviated with AD-F-L and AD-C-L in the AD-induced rats in the present study. Therefore, maintaining glucose homeostasis can prevent and mitigate AD impairment. Furthermore, the low-MW and high-MW fucoidan and λ-carrageenan of cognitive function are better conducted in the animals infused with full-length Aβ(1–40 or 1–42). 

Recently, SCFAs, the primary metabolites from the fermentation of dietary fiber by the gut microbiota, have attracted considerable interest for their role in AD pathogenesis [2]. Although AD pathogenesis is not completely understood, one potential mechanism is the Aβ deposition in the brain, particularly the hippocampus [35,53]. In previous studies, injecting Aβ into the hippocampal CA1 region of the animals stimulates hyperphosphorylated tau protein, a critical factor in the formation of neurofibrillary tangles, leading to a concomitant cognitive dysfunction [54,55]. The animals exhibited symptoms similar to those in humans with AD. The present study showed that AD-Con < AD-C-H < AD-C-L < AD-F-H < AD-F-L reduced hippocampal Aβ accumulation by enhancing the phosphorylation of Akt, GSK-3β, and STAT3, related to insulin/IGF-1 signaling. Furthermore, AD-F-L potentiated more STAT3, GSK-3β, and Akt phosphorylation than the other groups involved in the insulin/IGF-1 signaling pathway. Therefore, improvement of memory function by AD-F-L was linked to the potentiated hippocampal insulin signaling in AD-induced rats. 

Dysregulation of the human gut microbiota may be related to AD pathogenesis [56]. The α-diversity decline and SCFA changes in gut microbiota are detected in AD patients [2]. SCFAs are the predominant products of the gut microbiota that can be absorbed into the blood circulation from the intestines [2] and pass through the brain−blood barrier (BBB) and enter the brain [57]. They are the primary source for the gut−brain axis by stimulating the vagus nerve and can be used as an energy source instead of glucose in an insulin-resistant condition. SCFAs are potentially linked to Aβ accumulation in the brain [58]. Neurotrophic factors, such as BDNF, CNTF, and nerve growth factors, play crucial roles in protecting neurons and improving memory [59]. The BDNF and CNTF contents in the brain are closely related to the gut microbiota composition and SCFAs [13,60,61]. Germ-free mice have a lower hippocampal, amygdala, and cortex, and their fecal transfection increase BDNF contents to improve cognitive behavior [62]. However, the roles of SCFAs are controversial. In particular, acetate has been reported to increase insulin resistance and act as a proinflammatory factor to exacerbate memory impairment [58]. On the other hand, acetate is reported to have neuroprotective activity [59]. In the present study, the SCFA concentrations, including acetate, propionate, and butyrate, were lower in the AD-Con group than in the Normal-Con group. Serial acetic and butyric acid concentrations increased in the AD-F-L group compared to the AD-Con group. In one study, patients with mild cognitive impairment had lower serum SCFA levels than healthy controls, and AD patients had the lowest levels. The results indirectly support the close association between the SCFAs, consistent with the experimental results. Evidence suggests that AD is associated with gut microbiota disturbances, reducing chronic inflammation and neuronal function [63]. 

Fucoidan is a dietary fiber that influences the gut microbiota and potentially acts as a prebiotic, but it is an emerging research field. The size of fucoidans has not been studied. Previous studies have shown that fucoidan with a molecular weight of 310–1614 kD increased the abundance of probiotic bacteria *Lactobacillus* and *Akkermentia* and decreased the pathogenic bacteria, *Peptococcus*, *Helicobacter rodentium*, *Mucispirillum schaedleri*, *Rikenellaceae*, and *Alistipes* [64]. The results were partially consistent with the present study. The present study also showed that fucoidan with low (185 D–2.67 kD) and high (89–974 kD) molecular weights elevated the beneficial bacteria, *Akkermentia*, and decreased harmful bacteria, *Terrisporobacter*, *Phocaeicola*, *Sporofaciens*, *Mucispirillum*. Low-MW fucoidan intake elevated *Akkermentia* more than high-MW fucoidan. Therefore, smaller-size fucoidan potentially acts as a better prebiotic.

λ-Carrageenan is widely used as a thickener, emulsifier, and stabilizer, but it was reported to stimulate the innate immunity, gut microbiota composition, and thickness of mucus barrier. On the other hand, the effect of λ-carrageenan on gut microbiota and inflammation is unknown [65]. The present study showed that λ-carrageenan intake, regardless of the MW, improved memory function and gut microbiota to reduce secondary bile acid biosynthesis degradation of toxic compounds. Therefore, λ-carrageenan, regardless of MW, improved the gut microbiota to increase butyric acid production and decrease poisonous compounds. 

## 5. Conclusions

AD-F-L and AD-C-L improved the memory function, and AD-F-L potentiated it to levels similar to those in the Normal-Con group. AD-F-L potentiated both hippocampal insulin signaling and the expression of CNTF and BDNF in the brain, but AD-C-L improved memory function mainly by increasing the BDNF content. Compared to AD-Con, AD-C-L and AD-F-L increased insulin signaling by enhancing the pSTAT3→pAkt→pGSK-3β pathway. AD-F-L also improved glucose tolerance the most. Compared to AD-Con, AD-F-L increased the serum concentrations of acetate and butyrate to compensate for the defect in the cerebral glucose metabolism. AD-Con increased *Clostridium*, *Terrisporobacter*, and *Sporofaciens* compared to the Normal-Con group, and AD-F-L and AD-C-L increased *Akkermentia*. These results suggest that AD-F-L and AD-C-L alleviated memory function in rats with induced AD symptoms by modulating the glucose metabolism and gut microbiota. Fucoidan and λ-carrageenan, particularly small MW, are potential therapeutic options for preventing and delaying the progression of memory impairment. 

## Figures and Tables

**Table 1:** Body composition and food intake at the end of experimental periods.

	AD-Con	AD-F-H	AD-F-L	AD-C-H	AD-C-L	Normal-Con
Final weight (g)	410 ± 14.7	420 ± 11.5	426 ± 7.5 *	416 ± 11.4	428 ± 11.1 *	425 ± 8.9 *
Weight gain (g)	153.8 ± 7.9	168.6 ± 8.5	171.8 ± 9.0 *	159.6 ± 10.1	178.8 ± 8.3 *	174.6 ± 6.1 *
Food efficiency (%)	8.6 ± 0.4	9.1 ± 0.3	9.5 ± 0.5 *	8.3 ± 0.6	10.2 ± 0.4 *	9.8 ± 0.4 *
Epididymal fat (g)	7.49 ± 0.87	7.30 ± 0.51	7.72 ± 0.48	7.09 ± 0.60	7.49 ± 0.67	7.24 ± 0.54
Retroperitoneal fat (g)	8.47 ± 0.74	8.60 ± 0.65	9.02 ± 0.78 ^†^	7.67 ± 0.72	9.47 ± 0.60 ^†^	8.53 ± 0.60
Total visceral fat (g)	16.0 ± 1.56	15.9 ± 1.11	16.8 ± 1.20 ^†^	14.8 ± 1.20	17.0 ± 1.22 ^†^	15.8 ± 1.04

Food efficiency = food intake/weight gain * 100. Total visceral fat mass = epididymal fat + retroperitoneal fat. Values represent the means ± standard deviations (*n* = 10). * Significant from the AD-con group at *p* < 0.05. ^†^ Significant from the AD-F-L group at *p* < 0.05.

**Table 2:** Serum AST and ALT concentrations and triglyceride and glycogen contents in the liver and brain.

	AD-Con	AD-F-H	AD-F-L	AD-C-H	AD-C-L	Normal-Con
Serum AST (mg/dL)	68.1 ± 8.73	56.4 ± 5.73 *	51.1 ± 2.71 **	58.3 ± 3.63 *	60.8 ± 3.49	50.8 ± 4.32 **
Serum ALT (mg/dL)	9.34 ± 1.40	8.53 ± 1.47	7.23 ± 1.11 *	8.79 ± 1.16	8.76 ± 1.35	6.97 ± 0.68 *
Liver TG (mg/dL)	350 ± 8.58	337 ± 7.14 *	332 ± 9.67 *	342 ± 9.52	349 ± 6.16 *	342 ± 5.30
Liver glycogen (mg/dL)	89.0 ± 1.14	92.1 ± 1.33	101 ± 4.98	87.4 ± 1.48	110 ± 9.63 *	125 ± 6.32 **
Brain TG (mg/dL)	330 ± 18.8	345 ± 10.7	313 ± 15.0 ^‡^	337 ± 4.97 ^‡^	329 ± 25.2 ^‡^	356 ± 12.0
Brain TC (mg/dL)	2.28 ± 0.23	1.75 ± 0.13 *	2.02 ± 0.06	1.77 ± 0.10 *	1.79 ± 0.09 *	2.04 ± 0.11
Brain glycogen (mg/dL)	69.2 ± 5.39	78.2 ± 0.82 *	79.3 ± 0.40 *	79.3 ± 0.95 *	76.6 ± 4.11 *	95.3 ± 6.81 **

AST, aspartate aminotransferase; ALT, alanine aminotransferase; TG, triglyceride; TC, total cholesterol. Values represent the means ± standard deviations (*n* = 10). * Significant from the AD-Con group at *p* < 0.05 and ** at *p* < 0.01. ^‡^ Significant from the Normal-Con group at *p* < 0.05.

**Table 3:** Short-chain fatty acid (SCFA) concentration in the serum from the portal vein.

	AD-Con	AD-F-H	AD-F-L	AD-C-H	AD-C-L	Normal-Con
Acetic acid (mM)	0.67 ± 0.01	0.69 ± 0.001	0.74 ± 0.02 *	0.70 ± 0.008	0.72 ± 0.02	0.73 ± 0.03 *
Propionic acid (mM)	0.32 ± 0.001	0.32 ± 0.001	0.32 ± 0.001	0.32 ± 0.001	0.32 ± 0.001	0.32 ± 0.001
Butyric acid (mM)	0.16 ± 0.001	0.16 ± 0.001	0.17 ± 0.003 *	0.17 ± 0.002 *	0.17 ± 0.002 *	0.16 ± 0.001 *
Total SCFA (mM)	1.16 ± 0.01	1.17 ± 0.003	1.22 ± 0.02 *	1.19 ± 0.19	1.20 ± 0.02 *	1.21 ± 0.03 *

Values represent the means ± standard deviations (*n* = 10). Total SCFA was the sum of acetic acid, propionic acid, and butyric acid concentrations. * Significant from the AD-Con group at *p* < 0.05.

## Data Availability

The data presented in this study are available upon request from the corresponding author.

## Supplementary Materials

The following are available online at https://www.mdpi.com/article/10.3390/cells11152301/s1. Figure S1: Molecular weight of fucoidan and lambda-carrageenan measured using Gel Permeation Chromatograph/Preparative Gel Permeation Chromatograph.
