# Supplementary material for: Mitigation of Memory Impairment with Fermented Fucoidan and λ-Carrageenan Supplementation through Modulating the Gut Microbiota and Their Metagenome Function in Hippocampal Amyloid-β Infused Rats
Source: Cells. 2022 Jul 26;11(15):2301. doi: 10.3390/cells11152301 (PMC9367263; doi:10.3390/cells11152301)
Supplement: Supplementary file 1 [file cells-11-02301-s001.zip › cells-1788772-supplementary.pdf]

Supplementary Figure S1. Molecular weight of fucoidan and lambda-carrageenan measured using Gel Permeation Chromatograph/Preparative Gel Permeation Chromatograph

A. Chromatogram of fucoidan with no fermentation and its molecular size

B. Chromatogram of fucoidan with *Luteolibacter algae* and its molecular size

C. Chromatogram of lambda-carrageenan with no fermentation and its molecular size

D. Chromatogram of lambda-carrageenan with *Pseudoalteromonas carrageenovora* and its molecular size

A.

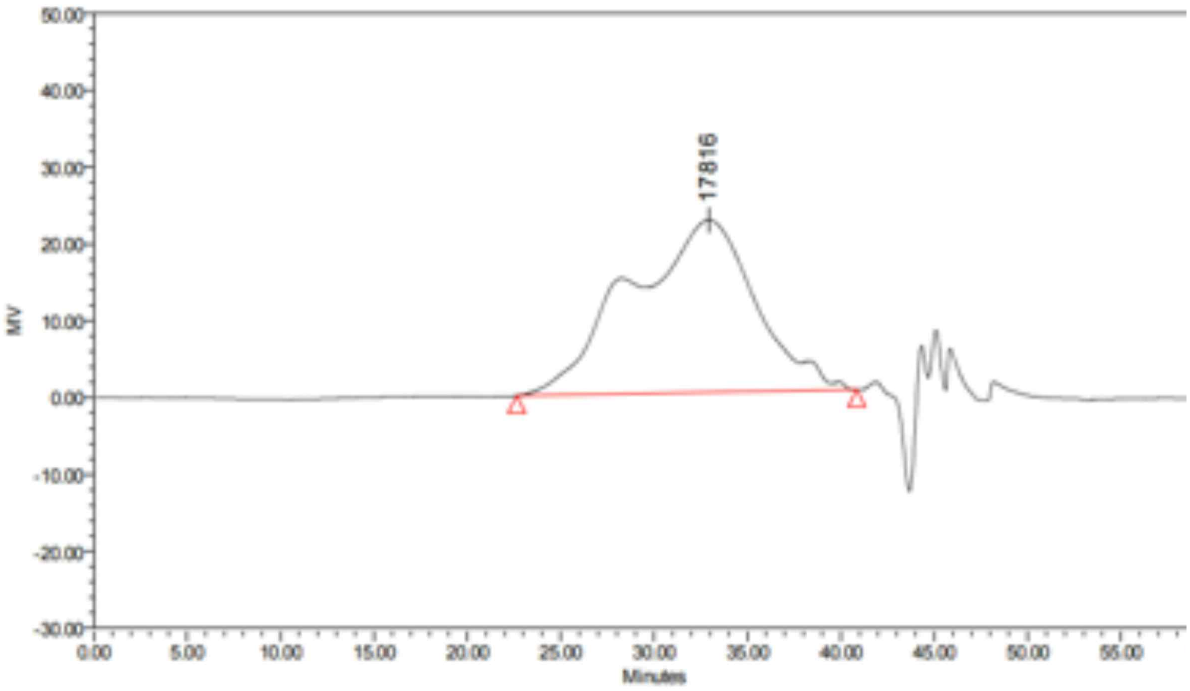

GPC Sample Results

	SampleName	Mn	Mw	MP	Polydispersity	% Area
1	Sample 1	7750	88959	17816	11.479	100.00

B.

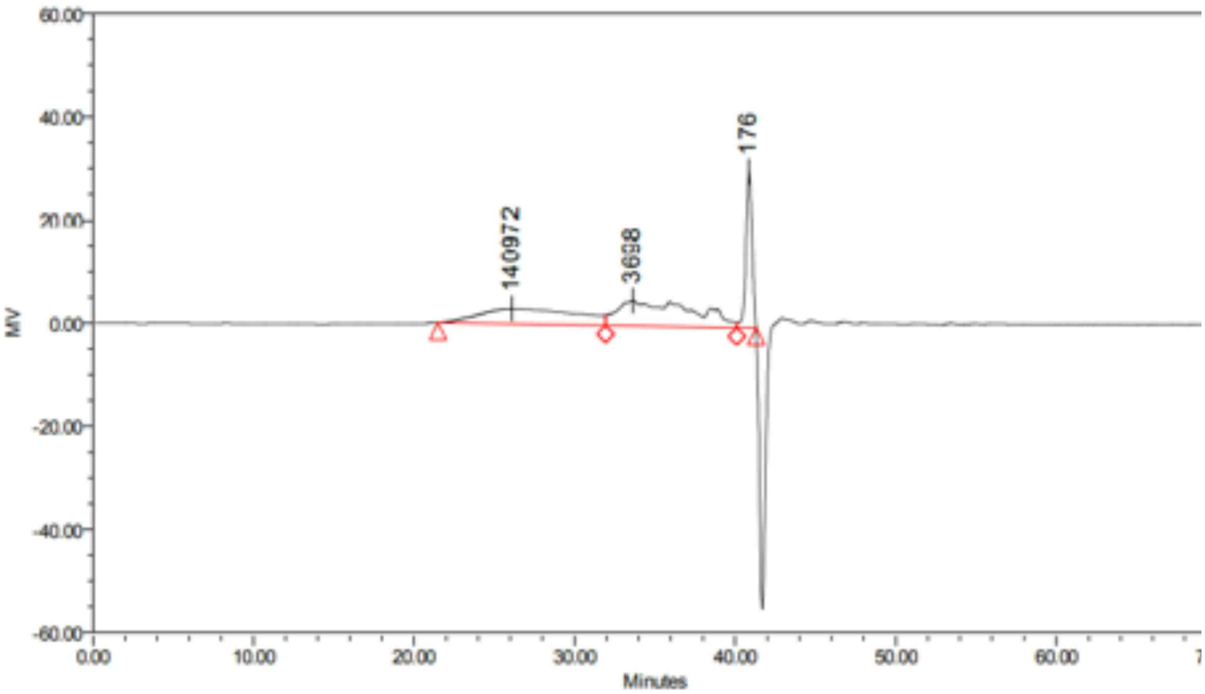

GPC Sample Results

	SampleName	Mn	Mw	MP	Polydispersity	% Area
1	Sample 6	39296	139315	140972	3.545	34.20
2	Sample 6	1014	2260	3698	2.227	43.65
3	Sample 6	177	178	176	1.005	22.14

C.

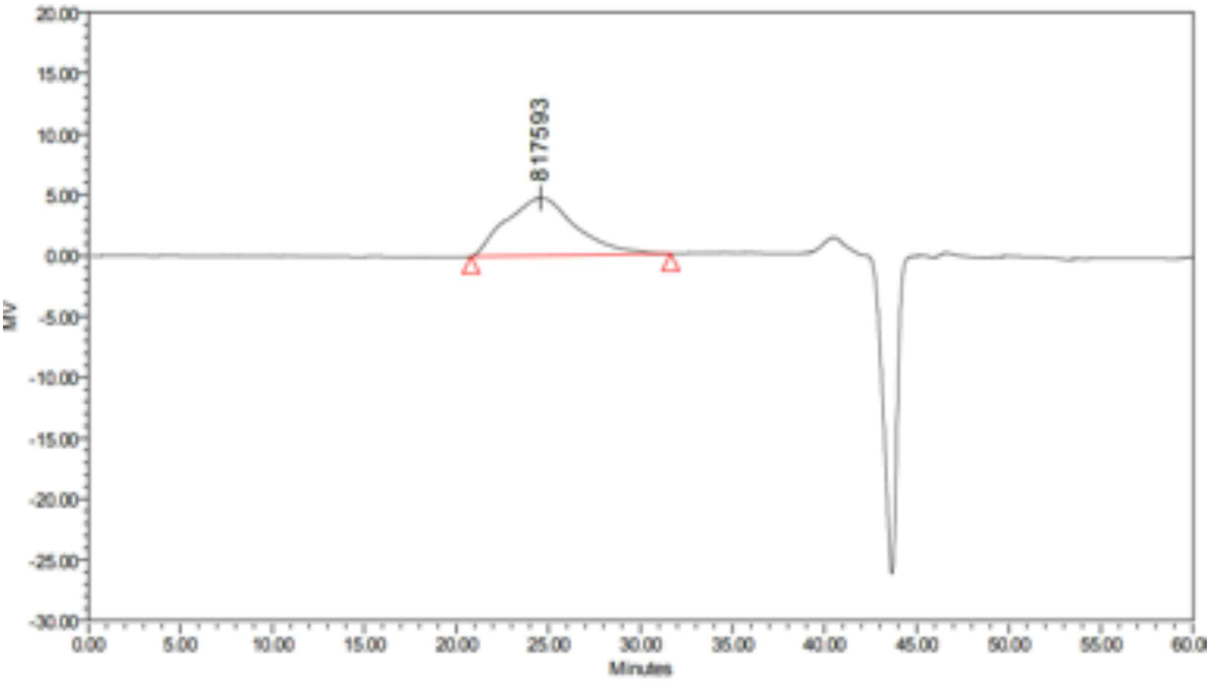

GPC Sample Results

	SampleName	Mn	Mw	MP	Polydispersity	% Area
1	Sample 2	529651	974600	817593	1.840	100.00

D.

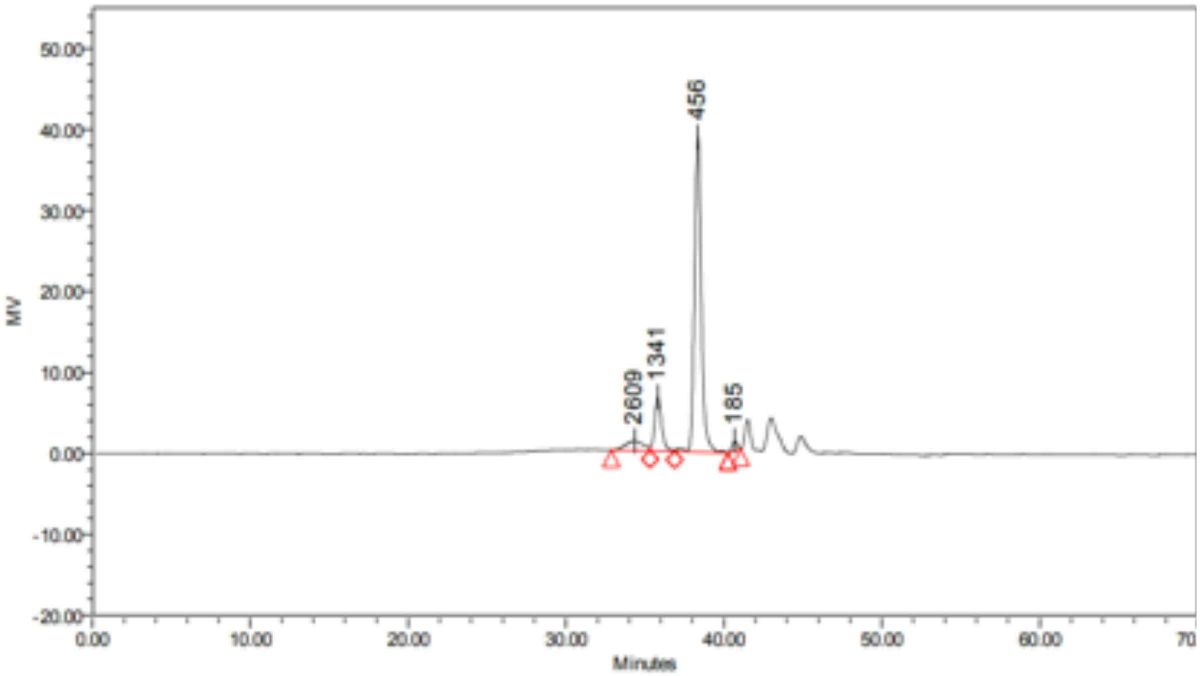

GPC Sample Results

	SampleName	Mn	Mw	MP	Polydispersity	% Area
1	C-late	2542	2672	2609	1.051	6.11
2	C-late	1303	1319	1341	1.012	13.68
3	C-late	449	456	456	1.013	78.38
4	C-late	185	185	185	1.003	1.83
